# Attitude, knowledge and informed choice towards prenatal screening for Down Syndrome: a cross-sectional study

**DOI:** 10.1186/s12884-018-2077-6

**Published:** 2018-11-12

**Authors:** Melania Elena Pop-Tudose, Dana Popescu-Spineni, Petru Armean, Ioan Victor Pop

**Affiliations:** 1 Department of Medical Genetics, “Iuliu Haţieganu” University of Medicine and Pharmacy, Pasteur Louis Street No.6, 400349 Cluj-Napoca, Romania; 2 Department of Specific Disciplines, Faculty of Midwifery and Nursing, “Carol Davila” University of Medicine and Pharmacy, Bucharest, Romania; 3 “Francisc I. Rainer” Anthropology Research Centre, Bucharest, Romania

**Keywords:** Attitude, Down syndrome, Informed consent, Knowledge, Prenatal screening

## Abstract

**Background:**

Down Syndrome screening test is a bridge between knowledge and uncertainty, safety and risk, unpredictability and desire to know in order to gain control. It may be accepted either not to have a baby with Down syndrome, or to prepare to have a baby with this condition. Every woman should understand that it is an option and should be encouraged to make their own decisions based on information and personal values. The implications and possible subsequent scenarios differentiate this type of test from the common biochemical tests performed during pregnancy, of paramount importance being the right to make informed choices. The aim of this study was to investigate the knowledge and attitude towards prenatal Down syndrome screening in order to asses to what extent the Romanian women make informed choices in this area.

**Methods:**

A cross-sectional study was carried out that included 530 postpartum women, clients of Romania’ south-east region maternities, during April–September 2016. The level of knowledge and the attitude concerning the Down syndrome screening were evaluated using a questionnaire. Data were analyzed using SPSS version 20.0.

**Results:**

48.1% of the women have never heard about any tests for Down Syndrome and from those 51.9% who have heard, only 14.2% made an informed choice, 78.9% had a positive attitude for screening, 88% were classified as having insufficient knowledge and 68.3% made a value-consistent decision to accept or decline prenatal screening. A higher knowledge level was associated with a higher education level and the urban residence. The information satisfaction and confidence in the overall value of screening were predictive factors of positive attitude. More informed choices were made by women monitored by an obstetrician in a private practice.

**Conclusions:**

The prenatal screening tests for Down Syndrome were mostly unknown and the women who accepted or not to perform a test were insufficiently knowledgeable that means that the ethical concept of the informed choice wasn’t followed. In our opinion the Romanian Health System needs to improve the antenatal policy by developing an adequate information strategy at the reproductive population level based on a network of trained specialists.

**Electronic supplementary material:**

The online version of this article (10.1186/s12884-018-2077-6) contains supplementary material, which is available to authorized users.

## Background

The incidence of Down Syndrome (DS) or trisomy 21 [1] is 1 in 650 to 1000 live births, representing the most common genetic cause of mental retardation (moderate to severe), the most common chromosomal disease of the newborn, but also the most compatible with survival of all autosomal trisomies. It is characterized by a particular facial aspect and may associate organic malformations, often cardiac. Persons with DS who benefit from special care may be socially integrated and live more than 60 years [2, 3]. Taking into account that it is caused mainly by a meiotic accident, all pregnant women have the risk of delivering a DS baby, a risk that increases steeply with maternal age [2]. Starting with 2007 the American College of Obstetrics and Gynecology (ACOG) recommends that screening for DS (DSS) to be available for all pregnant women regardless of age [4].

There is a wide range of DSS tests, with rates of prediction obtained either with a single test or combinations of several, thus offering multiple options. The latest technological advancement is Non-invasive Prenatal Test (NIPT), an investigation based on the analysis of free circulating fetal DNA in the maternal blood. It has a high predictability potential, that recommends it as a next in line screening in case of a positive traditional DSS, in order to avoid invasive methods [5, 6].

In Romania prenatal care is free of charge and it is part of primary care performed by family physicians. The Romanian health system refuses to include the midwife in the prenatal care team and the obstetricians can monitor healthy pregnant women only in private units. Traditional screening tests applied in Romania in the 1st trimester are the double test and/or the combined test (weeks 11–14 of amenorrhea), in the 2nd trimester the triple (between weeks 15–20) test and pregnancy morphology. These tests are not covered financially by the state and they are not part of the basic prenatal care package.

DSS identifies a condition that has no treatment yet, therefore deprived of the concept of prevention, which leads to a different meaning and multiple implications that require an altogether different approach [7]. In many countries the traditional DSS has become a routine practice [8, 9], attracting criticism because of its use by obstetricians as a “simple blood test”, performed without an informed consent [10]. Studies have shown a manipulation tendency by specialists in the sense of test acceptance, by infusing a feeling of responsibility for the normal development of the fetus [11, 12]. Sometimes the manipulation is pushed to the extreme by presenting DSS as mandatory without any information, mostly under the pressure of time [13]. The very presentation of the test as routine is manipulative, as it determines its perception and acceptance as a standard [14]. Even worse, the pregnant women often let to their obstetrician the choice of tests to be performed [12], based on the assumption that all of them are for their own good and interest, with scientifically documented benefits [15].

The main argument in favor of DSS is that is offers the possibility of an informed choice by the future parents [11]. The concept of informed choice in DSS context is based on both relevant knowledge and personal beliefs and values, all reflected in the behavior toward this type of test [16, 17]. The level and the amount of information provide the basis of autonomy, it marks and orients the choice [13]. Providing biased, inaccurate, incomplete or insufficient information, either deliberately, because of haste, ignorance, or to protect the mother, is against all ethical principles, with devastating consequences over time [7, 15, 18, 19]. This prompts for the necessity to present DSS in full and without bias, and also DS in all its aspects, negative and positive, with the implications and progress up to date [20–22], in a manner adapted to the level of education and the social-spiritual profile of the audience [11]. Obviously, such a presentation cannot be a stereotype but individualized, which requires time and specialists trained to deliver such information [19, 23]. Most studies show that in fact the pregnant women are not sufficiently informed and have limited knowledge about the aspects of the DSS process (the majority does not have the basic knowledge) and very rarely their beliefs, personal values, preferences and their need to deliberate are taken into account [9, 24–26].

The decision to take DSS is important and hard, given the possible scenarios, the probabilistic uncertainty and the impossibility to anticipate or guarantee results [23]. Both the decision and the choice of the type of testing should belong to woman or to both parents and not to local policies or to the doctor [24]. The range of options should also include the option of no testing [23, 27]. The ethics of DSS will obviously depend on the social policy and context [11].

Unfortunately the policies and screening programs influence DSS absorption [8, 12], many countries aiming at maximizing absorption, not informed absorption though, which justifies the questions raised: does DSS serve its purpose, does it observe informed choice, or not? [27]. This study started from the hypothesis that those women who have heard about at least one DSS test have a positive attitude but most probably have insufficient knowledge in order to make informed choices.

## Methods

This was a cross-sectional study, carried out in 7 maternities of the South East region of Romania, during April–September 2016. Questionnaires were administered to the postpartum women on days 3–4 after delivery and included 4 sections: attitude and information satisfaction (18 items), knowledge (24 items), demographic data (7 items), as well as data related to pregnancy follow-up (7 items) (Additional file 1). The items used were adapted from two previous studies Rostant et al. [13] and Pruksanusak et al. [28] that have been validated in Australia and Thailand. Reliability of the knowledge and attitude measures were assessed on a group of 20 volunteers by Cronbach’s alpha coefficient and we obtained 0.82 for knowledge scale and 0.87 for the attitude scale. We excluded women with cognitive deficits or other conditions that would prevent them from understanding the nature and purpose of the study and/or from providing requested information, as well as mothers whose babies were premature, had died, or had impaired health. Every questionnaire was accompanied by an information sheet and filling the questionnaire was considered a consent to participate. For participants under age of 16, verbal informed consent was obtained from one of their parents and the parental consent was witness by the institution’s staff. The questionnaires were handed out personally by the main researcher to 610 postpartum women of which: 80 returned an unfilled questionnaire, meaning a refusal to participate, 530 (76.4%) accepted to fill in but 255 (48.1%) of them had never heard about any DSS tests and answered partially (demographic and follow up questions) and only 275 (51.9%) heard about them and completed the whole questionnaire. Data were processed with the IBM SPSS 20.0 (IBM Corp., Armonk, NY, USA). We checked for normal distribution using Chi-Square Test, Kolmogorov-Smirnov and Shapiro-Wilk tests, we applied Spearman’s Rank-Order Correlation, Cross Tabulation, Mann-Whitney U and Kruskal Wallis H tests and we performed PCA (Principal Component Analyses) for the attitude and informational satisfaction scale. Statistical significance was set at *p* < 0.05. Knowledge was measured by a scale consisting of 24 items. The scale was based on answers of “yes”, “no” and “do not know” about these tests. An answer showing accurate knowledge (which can be yes or no, depending on the question) received 1 point and an answer showing the lack of knowledge or “do not know” was scored with 0 points. The women with a total score of equal or less than 8 were classified as having a low level of knowledge, the women with a score between 9 and 16 were classified as having an average level and the women with a score between 17 and 24 were classified as having a high level of knowledge. We have taken into account the fact that in order to make an informed choice, it is necessary to have a level of knowledge above average. Data obtained through surveys of attitude, based on likert scale, were divided into 3 categories acording to the scores obtained: negative attitude (disagreement), neutral, and positive attitude (agreement). We also analyzed the concordance between attitude and survey behavior (consistency value), and the existence of informed choice. For consistency the van den Berg et al. [26] model was used, assessing the concordance of behavior (taking, not taking the survey) with attitude (positive, negative). For the assessment of informed choice we made the correlation between the value consistency and the level of knowledge after the same model.

## Results

Initially, data were collected from 530 post-partum women but after we had analyzed the questionnaires we divided the participants into two groups: Group I included 255 (48.1%) women who had never heard about any DSS and from whom we received only demographic and follow up data and the Group II with 275 (51.9%) women who had heard of at least one DSS. The mean age of the Group I was 25.91 years (SD = 6.51, range 14 to 43), three-quarters of them were from rural areas and had a low level of education and about a quarter of them were Roma (Table 1). Only half of them had been followed-up by an obstetrician (nearly a quarter alternatively with a familly doctor), but less than a quarter of them did not follow-up their pregnancy with anyone and only less than a quarter had a private care (Table 2).

**Table 1:** Demographic characteristics (*N* = 530)

Category	Group I *N* = 255		Group II *N* = 275	
	Number %		Number %	
Age				
14–24	114	44.7	78	28.4
25–34	113	44.3	167	60.17
35–44	28	11	30	10.9
Ethnicity				
Romanian	189	74.1	268	97.5
Roma	61	23.9	5	1.8
Other	5	2	2	0.7
Religion				
Christian	241	94.5	268	97.5
Other	14	5.5	7	2.5
Education level				
Low	187	73.3	61	22.2
Medium	63	24.7	147	53.5
High	5	2	67	24.3
Residence				
Urban	74	29	149	54.2
Rural	181	71	126	45.8
No. of children				
1	87	34.1	153	55.6
2	75	29.4	98	35.6
≥3	93	36.5	24	8.7
Abortion/miscarriage				
Yes	105	41.2	104	37.2
No	150	58.8	171	62.2

**Table 2:** Data related to follow-up and information (*N* = 530)

Category	Group I *N* = 255		Group II *N* = 275	
	Number %		Number %	
Follow-up				
Family doctor	78	30.6	23	8.4
Obstetrician	75	29.4	134	48.9
Both	49	19.2	118	48.7
None	53	20.8	0	0
Where				
Private Services	33	12.9	116	42.2
State Services	146	57.3	82	29.8
Both	23	9	77	28
None	53	20.8	0	0
Time allocated by the specialist for DSS presentation				
0 min	N/A	N/A	27	9.8
≤10 min	N/A	N/A	169	61.5
15-20 min	N/A	N/A	37	13.5
≥30 min	N/A	N/A	43	15.3
Using teaching aids				
Yes	N/A	N/A	49	17.8
No	N/A	N/A	226	82.2
Took at least one test				
Yes	N/A	N/A	149	17.8
No	N/A	N/A	126	82.2

Because the study’s aim was to investigate the knowledge, attitude and informed choice towards prenatal DSS, we concentrated our research on Group II, who had heard of at least one screening test.

The mean age of the Group II was 27.88 years (SD = 5.45, range 15 to 44). More than three quarters of the women (*N* = 221, 80.4%) had undergone one or more tests, and less than one quarter (*N* = 54, 19.6%) were not tested. Overall, almost all the respondents (*N* = 252, 97.6%) had been followed up by an obstetrician, half of them only by the obstetrician and the other half by obstetrician and family physician (alternatively). About three quarters of the women (*N* = 193, 70.2%) resorted to private medical services, more precisely half of them used only private services and about a quarter both private and state insurance services (alternatively). A small percentage appealed only to the family physician and more than a quarter used strictly state ensured services. For more than a half of the participants, the time allocated on presenting DSS was equal or less than 10 min. The average time of DSS presentation by specialists was 12.23 min (Median = 10, SD = 15.26, range 0 to 120 min).

### Women’s knowledge

The mean knowledge score was 10.9 (range 0 to 22, SD = 4.77). None of the respondents obtained the maximum score, whilst 2.9% (*N* = 8) had no knowledge of these tests. The classification by 3 levels of knowledge showed that more than half of the participants (*N* = 157, 57.1%) had an average level of knowledge about DSS, more than a quarter (*N* = 85, 30.9%) a low level, and only a small proportion (*N* = 33, 12%) a high level considered sufficient for an informed choice. Thus, starting from the concept that informed choice implies adequate information, above the average, we considered that 88% (*N* = 244) of the participants had insufficient knowledge to abide by this concept.

We found that knowledge increased significantly (χ2 = 49.51, df = 2, *p* < 0.001) by undergoing the DSS process, due to the higher level of general education (χ2 = 30,63, df = 4, *p* < 0.001), due to a follow-up with an obsterician (χ2 = 20.46, df = 2, *p* < 0.001), accessing private services (χ2 = 21.65, df = 2, *p* < 0.001) and increasing the time allocated to DSS presentation by specialists (χ2 = 10.31, df = 2, *p* = 0.006). We also found significant differences related to the living background, the urban participants had better knowledge than the rural ones (U = 8142, *p* < 0.05) and among the group given teaching aids (video, brochures, leaflets) (U = 4134, *p* = 0.002), that scored higher compared with those who had no visual or information materials. There was a positive correlation, though weak, between knowledge and women’s age (*r* = 0.12, < 0.05). We found no correlations with the number of live children or abortions/miscarriages. For a better comprehension of the differences related to the demographic and follow up variables, a comparison of the medians is given in Table 3.

**Table 3:** Median scores of knowledge survey related to demographic and follow-up variables (*N*^c^=275)

Variables	*N*	Median (IQR)	Test statistics	*p*-value
Took at least one test				
Yes	221	12(6)	2868.0^a^	0.000
No	54	6.5(5)		
Age				
14–24	78	10(7)		
25–34	167	12(7)	5.872^b^	0.050
35–44	30	12(6)		
Residence				
Urban	149	14(5)	8142.0^a^	0.032
Rural	126	9(5)		
Education level				
Low	61	8(6)	30.503^b^	0.000
Medium	120	11(7)		
High	55	14(4)		
Follow-up				
Family doctor	23	6(7)	22.489^b^	0.000
Obstetrician	134	12(6)		
Both	118	11(7)		
Where				
State services	82	8.5(7)	22.325^b^	0.000
Private services	116	13(6)		
Both	77	11(6)		
Teaching aids				
Yes	49	13(5)	4134.0^a^	0.002
No	226	11(7)		
Time allocated by the specialist for DSS presentation				
0 min	27	7(6)	24.820^a^	0.000
≤ 10 min	169	11(7)		
15–20 min	37	13(7)		
≥30 min	42	13(5)		

^a^Mann Whitney Test

^b^Kruskal Wallis Test (*p* ≤ 0.05)

^c^Women who heard about at least one DSS

### Women’s attitude

The mean attitude score was 20.36 (range 0 to 22, SD = 3.39). More than three quarters (*N* = 217, 78.95%) had a positive attitude to DSS, almost a quarter (*N* = 56, 20.4%) were neutral, and only 0.7% (*N* = 2) had a negative attitude. Attitude was not influenced significantly by any demographic variable, though mild correlations were found in relation to follow-up. Those women followed up in a private practice, by an obstetrician who allocated more time to presentations and explanations about DSS, were more positive (*r* = 0.18, *r* = 0.16, *r* = 0.12, *p* < 0.05). We found significant differences between those who underwent at least one DSS and those who did not (U = 4335, *p* < 0.001); the former had a more positive attitude. We investigated the relation between the knowledge level and the attitude of those who took at least one test. As Table 4 shows, the two variables were correlated (phi = 0.27), better knowledge slightly increasing the positive attitude tendency.

**Table 4:** The relationship between knowledge and attitude of those who took at least one DSS (*N*^a^=221)

Attitude			
Knowledge	Neutral *N* (%)	Positive *N* (%)	Test
Low	16 (7.2)	31 (14.2)	χ2 = 15.49, df = 2, *p* < 0.001
Medium	17 (7.7)	125 (56.5)	
High	2 (0.9)	30 (13.6)	
Total	35 (15.8)	186 (84.2)	

^a^No negative attitude was found in women who were submitted to at least one DSS

Further on we performed the PCA for attitude and informational satisfaction scale of the questionnaire completed by the women who had at least one DSS (*N* = 221). Three factors were initially extracted with an Eigen value ≥1. After oblim extraction and scatter plot analysis we chose only two factors that explained 55% of the variance. The first factor was the attitude towards the adequate information (39.8%) and the second the attitude towards the overall benefits of the test (15.2%).

### The value consistency and the informed choice

Starting from the fact that an informed choice is based on the level of knowledge of the subject and the personal values and beliefs reflected in the attitude and the behavior towards DSS, we searched for a concordance between the attitude toward DSS and the women’s behavior (taking or not the test), as well as the degree of informed choice. Summing up the number (N^a^) of participants whose behavior was according with their attitude (Table 5), we found that in almost three quarters (*N*^a^ = 188, 68.3%) the attitude rhymed with behavior. A neutral attitude cannot be taken into account when referring to informed consent, therefore neutral cases were excluded (*N* = 275–56 = 219). We found that only 14.2% (*N*^a^ = 31) of participants (Table 6), who were pro-active, made an informed choice, according with behavior and based on a sufficient level of knowledge, while more than three quarters (*N*^b^ = 188, 85.8%) made a choice without being informed.

**Table 5:** Attitude towards having DSS of the women who accepted/declined the test offer

Attitude	Acceptors *N* (%)	Decliners *N* (%)	Total *N* (%)
Positive	186^a^ (67.6)	31^b^ (11.3)	217 (78.9)
Neutral	35 (12.7)	21 (7.6)	56 (20.4)
Negative	0^b^ (0)	2^a^ (0.7)	2 (0.7)
Total	221 (79.5)	54 (20.5)	275 (100%)

^a^These categories represent value-consistent decisions

^b^These categories represent value-inconsistent decisions

**Table 6:** Value (in-) consistency and knowledge about DSS (*N*^c^=219)

Knowledge			
	Insufficient *N* (%)	Sufficient *N* (%)	Total *N* (%)
Value consistency	157^b^ (71.7)	31^a^ (14.2)	188 (85.8)
Value inconsistency	30^b^ (13.7)	1^b^ (0.5)	31 (14.2)
Total	187 (85.4)	32 (14.6)	219 (100%)

^a^These categories represent informed choice

^b^These categories represent uninformed choice

^c^without the neutral participants

We analyzed the two groups (informed and uninformed choice) in relation to their independent variables (demographic, follow-up, information). Significant differences were found between the two groups regarding the education level (H = 10.90, *p* = 0.004), the specialist (U = 5240.5, *p* < 0.05) and the health unit (U = 5331.5, *p* < 0.05) where they followed- up their pregnancy. However, no significant difference (*p* ≥ 0.05) was seen in terms of age, background, number of children or lost pregnancies, nor to the training length of time or teaching aids.

## Discussion

We didn’t expect to find so many women who had never heard about at least one DSS. Analyzing their demographic profile we can see that most of them came from rural places, had a low education and an inadequate follow-up in pregnancy. It’s a fact that suggests to us that there is a possible discrimination in terms of health care services accessing.

However, as we expected, over three quarters of women who had heard about at least one DSS, had a high positive attitude towards DSS, a result in line with other studies: Pruksanusak et al. [28], Gourounti and Sandall [16], Rostant et al. [13], and van den Berg et al. [26]. Moreover, our hypothesis was reinforced by the poor level of knowledge found, as few of the women succeeded in completing the knowledge test with high scores that would sustain an informed choice. In fact the analysis of informed choice, based on the knowledge level and value-consistent decisions, evidenced that only 14.2% of the pro-active study women could qualify as informed for making a choice.

Our study along with the Australian [13], Thai [28] and Greek [16] studies found a poor knowledge level, only the Dutch study [26], found that the majority of the participants had sufficient knowledge about DSS but this may be due to the fact that study participants received “an information booklet about DSS” before the administration of the questionnaire. There are a number of similarities between our results and those of the above-mentioned studies, but also differences regarding the possible influences. Our study as well as those by Rostant et al. and van den Berg et al. found a higher tendency to be positive in the women who had undergone at least one DSS. We could state, like Rostant et al., that this may be because those women benefited from additional information from specialists. More than that, like in the Australian study, the strongest predictive factor of positive attitude was the good information process. On the other hand, Rostant et al. and we identified a positive correlation between attitude, older age and higher education level, in contrast with Pruksanusak et al., who reported younger age and lower education as correlated with positive attitude. In our study, knowledge was positively correlated mainly with average to high level of general education, pregnancy monitored by a specialist, preferably in a private practice, a longer time allocated to DSS presentation, and also with age and teaching aids, handouts, especially the emphasis on explaining fully the DSS process. In the studies mentioned (except Gourounti and Sandall), higher levels of education and older age were also found positive for the knowledge process. Unlike in our study, Rostant et al. found that undergoing the whole DSS process was negatively associated with knowledge levels, but like us they found a positive association between knowledge level and private follow-up, use of teaching aids and urban background. For the women qualified as making an informed choice – 14.6% in our study, 44% in Gourounti and Sandall, and 68% in van den Berg et al. – the common predictors were the high level of education and a good financial status, the latter expressed in our study by access to private care, which is considered expensive in our country.

This study, the first in Romania on this topic, had the main objective to investigate the level of knowledge, the attitude and the degree of informed choice about DSS. Because nearly half of the participants had never heard about any DSS, the remained group for the investigation was smaller than we anticipated so that it cannot be considered representative for the region explored. However, our findings can represent a big warning for the authorities to rethink the prenatal care policies. We also consider that we’ve already opened a door for a future extensive study based on our methods that could investigate a large group of pregnant women to find out how well they are informed about all possible investigations in pregnancy.

## Conclusions

For those women who have never heard of DSS, we can talk about violating the human right to be informed and implicitly about obstructing the right to have a choice which can be a good subject for another research.

Furthermore, a mediocre level of knowledge, as we identified in those who heard about at least one DSS, is insufficient regarding to the concept of informed choice, and most probably the almost dominant positive attitude was related to maternal responsibility and the wish to fulfill it by accepting the investigations proposed.

On the other hand, the high rate of “don’t know” responses (41.1%) showed that the respondents did not want to give answers arbitrarily, nor they didn’t want to appear ignorant. In support of this statement is the fact that after questionnaire completion an impressive number of women asked for the correct answers and other additional information, which our researcher provided by handing out leaflets and informative materials. This suggests that women want to be informed and the usual practice to offer specific information in a routine obstetric consulting is not a proper one.

In conclusion, this study suggests that DSS is mostly unknown and women who accepted or not to perform a test were insufficiently knowledgeable which means that the ethical concept of the informed choice wasn’t followed. It also underlines the necessity of introducing DSS as an option on the list of the maternity insurance services. In our opinion, the Romanian healthcare system is subpar in this field and it is required to develop an information strategy for the reproductive population based on a network of trained specialists (midwives, genetic counselors) to provide women with adequate information to enable them to make an informed choice and facilitate their decision-making process.

## Additional file

Additional file 1: A survey of new mothers about screening for Down syndrome. Questionnaire given to new mothers about screening for Down syndrome. (DOC 215 kb)

## Abbreviations

ACOG: American College of Obstetrics and Gynecology
DNA: Deoxyribonucleic acid
DS: Down syndrome
DSS: Down’ syndrome screening
NIPT: Non-invasive Prenatal Test
PCA: Principal Component Analyses
SPSS: Statistical package for the social sciences
