# Supplementary material for: Attitude, knowledge and informed choice towards prenatal screening for Down Syndrome: a cross-sectional study
Source: BMC Pregnancy Childbirth. 2018 Nov 12;18:439. doi: 10.1186/s12884-018-2077-6 (PMC6233289; doi:10.1186/s12884-018-2077-6)
Supplement: Supplementary file 1 — A survey of new mothers about screening for Down syndrome. Questionnaire given to new mothers about screening for Down syndrome. (DOC 215 kb) [file 12884_2018_2077_MOESM1_ESM.doc]

**PRENATAL SCREENING**

**FOR**

**DOWN SYNDROME**


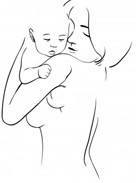


**A survey of New Mothers**


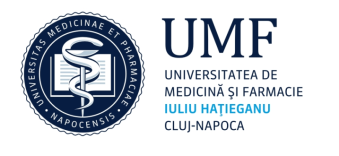


**The following survey relates to „ prenatal screening tests”, which are carried out during pregnancy to detect Down Syndrome.**

1. **Have you heard of ANY of these prenatal tests?**

Yes ⇒ Please continue

No ⇒ Please go to Question 5,6 on page 3 and Questions 51 to 57 on page 6

1. **Which of the following tests have you :**

(Please tick either the”Yes”, „No” or „Unsure” boxfor each part of the question):

| **Prenatal screening tests** | **Heard of** | **Actually had** |
| --- | --- | --- |
| Ultrasound and blood test before 11-13weeks/aprox. 3 month of pregnancy | Yes  No  Unsure | Yes  No  Unsure |
| Second trimester maternal serum screening (MSS) | Yes  No  Unsure | Yes  No  Unsure |
| Second trimester morphology ultrasound | Yes  No  Unsure | Yes  No  Unsure |
| Non-Invasive Prenatal Testing (NIPT) | Yes  No  Unsure | Yes  No  Unsure |

1. **Where did you first find out about the tests?** (Please tick ONE box Only)

Family doctor Previous pregnancies

Obstetrician Internet

Midwife Media

Family/Friends Pamphlets/resources

Other (Please specify)...................................................................

1. **Who/what provided you with most information about prenatal screening tests ?**

(Please rate your top 3)

Family doctor Previous pregnancies

Obstetrician Internet

Midwife Media

Family/Friends Pamphlets/resources

Other (Please specify).................................................................

1. **Who follow-up your pregnancy ?**

Family doctor Midwife
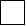


Obstetrician None
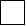


Other (Please specify )……………..............................................

1. **Where?** State Institution Private Institution

1. **Have you seen/received materials about Down Syndrome and prenatal testing for it?** (Please tick ONE box Only)

Yes

No

Unsure

**For the next questions please and indicate the extent to which you agree or disagree with the statement by circling the relevant number for each question (follow the example).**

| **Example** | |  | **Strongly Disagree** | | **Disagree** | **Neutral** | **Agree** | **Strongly**  **agree** | |
| --- | --- | --- | --- | --- | --- | --- | --- | --- | --- |
| Summer is the best time of year. | | | 1 | | 2 |  | 4 | | 5 |
| **Your Answer(s):** | | | | | | | | | |
| **8.** | Prenatal screening tests are valuable | | | 1 | 2 | 3 | 4 | | 5 |
| **9.** | Prenatal screening tests benefit all pregnant women | | | 1 | 2 | 3 | 4 | | 5 |
| **10.** | All pregnant women should have prenatal screening tests | | | 1 | 2 | 3 | 4 | | 5 |
| **11.** | The cost of prenatal screening tests should not influence whether they are done or not | | | 1 | 2 | 3 | 4 | | 5 |
| **12.** | If I were to have another pregnancy I would have prenatal screening tests | | | 1 | 2 | 3 | 4 | | 5 |
| **13.** | During my pregnancy I was worried about my baby health | | | 1 | 2 | 3 | 4 | | 5 |
| **14.** | Prenatal screening tests help reduce anxiety during pregnancy | | | 1 | 2 | 3 | 4 | | 5 |
| **15.** | It would be verry difficult for me if I had a child with Down Syndrome | | | 1 | 2 | 3 | 4 | | 5 |
| **16.** | During my pregnancy I was certain that my baby was healthy | | | 1 | 2 | 3 | 4 | | 5 |
| **17.** | The information found on internet or other unofficial sources made me worry about my baby | | | 1 | 2 | 3 | 4 | | 5 |
| **18.** | I had enough time to make a decision whether to have the test or not | | | 1 | 2 | 3 | 4 | | 5 |
| **19.** | I was provided with enough information about prenatal screening tests | | | 1 | 2 | 3 | 4 | | 5 |
| **20.** | The information I was given was clear | | | 1 | 2 | 3 | 4 | | 5 |
| **21.** | My doctor/midwife knew enough about the tests that were available | | | 1 | 2 | 3 | 4 | | 5 |
| **22.** | I was given enough information to help me make the decision to have the test. | | | 1 | 2 | 3 | 4 | | 5 |
| **23.** | The results of my tests were explained clearly | | | 1 | 2 | 3 | 4 | | 5 |
| **24.** | I felt confident about the results of my tests | | | 1 | 2 | 3 | 4 | | 5 |
| **25.** | I was given enough information about the test results | | | 1 | 2 | 3 | 4 | | 5 |

| **For each next statement please tick whether in your opinion that the statement is „true”, „false” or you "don't know”.** | | **True** | **False** | **Don’t Know** |
| --- | --- | --- | --- | --- |
| **26.** | Down syndrome (DS) is one of the most common birth defects, affecting about one in every 750 live births |  |  |  |
| **27.** | Down syndrome is a genetic defect that occurs at the conception of the baby |  |  |  |
| **28.** | All Down syndrome children have mental retardation |  |  |  |
| **29.** | Down syndrome children may have congenital structural abnormalities such as heart disease, gastrointestinal disease, etc. |  |  |  |
| **30.** | Down syndrome children need someone to take special care of them |  |  |  |
| **31.** | Down syndrome children could be trained |  |  |  |
| **32.** | Down syndrome fetuses have a higher chance of abortion than normal ones. |  |  |  |
| **33.** | All pregnant women have a chance of having a Down syndrome fetus |  |  |  |
| **34.** | Risk of having Down syndrome fetus is higher as maternal age advances |  |  |  |
| **35.** | If there is no case of Down syndrome in the woman's and her partner's family means that her risk of giving birth to a child with this syndrome is zero |  |  |  |
| **36.** | First trimester screening involves ultrasound and maternal blood test |  |  |  |
| **37.** | The Down syndrome screening tests only tell us that the fetus has more or less chance of having Down syndrome. |  |  |  |
| **38.** | Tests can be done as early as 11-13 weeks/aproximately 3 months to identify pregnancies at risk of Down Syndrome |  |  |  |
| **39.** | Second trimester maternal serum screening can be done at 15-20 weeks/aproximately 5 months to identify pregnancies at risk of Down Syndrome |  |  |  |
| **40.** | Following a screen test, 5% (1 in 5) of women receive an **at increased risk** result |  |  |  |
| **41.** | Most women (98%) who receive an at increased risk result have healthy babies |  |  |  |
| **42.** | Ultrasound can detect all cases of Down Syndrome |  |  |  |
| **43.** | If a positive or at increased risk result is given this mean the fetus definitely has Down Syndrome |  |  |  |
| **44.** | Women who had normal screening results can be certain that they will have a healthy baby |  |  |  |
| **45.** | If the screening test shows at increased risk, further tests can be done to clarify a diagnosis |  |  |  |
| **46.** | NIPT* is a maternal blood test |  |  |  |
| **47.** | NIPT* can be done early in pregnancy, beginning with 9-10 weeks |  |  |  |
| **48.** | NIPT* has a detection rate of over 99% for Down syndrome |  |  |  |
| **49.** | A positive NIPT* result should always be confirmed with invasive testing |  |  |  |

*Non-Invasive Prenatal Testing (**NIPT**)

**50. Please, approximate the time (in minutes) spent with your specialist talking about prenatal testing**

**51. What is your year of birth?.........**

**52. How many children do you have?..................................**

✂

**53. Did you have any misscariage/abortion?......... If Yes How many?.......**

**54. What is the highest level of education you have completed?** (Please tick ONE box only)

Primary (Year 4) Vocational School

Secondary(Year 8) Post-High school

Year 10 University

High school (Year 12) Post-University

**55. Which ethnic group do you belong to? ......................................**

**56. Which religion group do you belong to?......................................**

**57. Where you live?**

City

Rural area

**Thank you for taking the time to complete this survey!**

**INFORMATION FORM**

You are invited to take part in a research study. For this we ask you to complete a questionnaire which will take 15-20 minutes. Before you decide to participate, it is important for you to understand the reason for carrying out this study, what involves it and what benefits and risks can bring to you. Please read carefully the following information and if you have further questions about the study or about the completing procedure, please address to the investigator before begin to complete the questionnaire.

**What is this all about ?**

The study is run under the aegis of the Doctoral School of the "Iuliu Hatieganu" University of Medicine, Cluj-Napoca, and the aims are to obtain a realistic perspective of the awareness, experience and availability of information among women in Romania on prenatal testing for Down Syndrome.

**Is it mandatory to participate?**

You decide whether to participate or not. If you decide to participate we want you to know you are free to withdraw from the study at any time without the need for any justification. This or the decision to not participate will not affect the quality of health care that you receive within this medical institution.

**What about my confidentiality?**

Since this questionnaire does not contain name and address details and required demographic data can not disclose the identity of the participant, we assure that the study is run under the aegis of anonymity and it does not involve any risk to you. This means that your replies will be used to achieve the intended purpose, and the data obtained may be published or presented at conferences, but your and other participants identity will remain anonymous.

**What are the possible benefits of participating in this study?**

Maybe for you there are no direct benefits, at this time, but in our opinion the information obtained through this study could be the basis of developing future health policies for a better services in this field.

**Thank you for taking the time to read this document.**

**Investigator**

Melania Elena Pop-Tudose – tel.0722 751 540

melaniaelena_tudose@yahoo.ro
